# Martensitic transformation of Ti_50_(Ni_50−*x*_Cu_*x*_) and Ni_50_(Ti_50−*x*_Zr_*x*_) shape-memory alloys

**DOI:** 10.1038/s41598-019-40100-z

**Published:** 2019-03-01

**Authors:** Xiaolan Yang, Lei Ma, Jiaxiang Shang

**Affiliations:** 1 School of Materials Science and Engineering, Beihang University, Beijing, 100191 China; 2 School of Physics and Electronic Science, Zunyi Normal College, Zunyi, 563002 China

## Abstract

Martensitic transformation and phase stability of Ti_50_(Ni_50−*x*_Cu_*x*_) and Ni_50_(Ti_50−*x*_Zr_*x*_) shape memory alloys are investigated based on density functional theory (DFT). According to the results of formation energy we calculated, upon substitution of Ni by Cu at levels of about 10.4 at.%, Ti_50_(Ni_50−*x*_Cu_*x*_) alloys lose the monoclinic martensite in favor of the orthorhombic martensite structure. The martensite monoclinic B19´ structure of Ni_50_(Ti_50−*x*_Zr_*x*_) becomes more stable with increasing of the Zr content. The energy difference between austenite and martensite decreases when Cu < 10.4 at.%, and then increases slightly, which suggesting that Cu addition reduces the composition sensitivity of martensitic transformation temperature comparing with binary NiTi alloys. The energy difference decreases slightly firstly when Zr < 10.4 at.% and then increases sharply, which indicates that Zr addition increases martensitic transformation temperature dramatically. Furthermore, a geometric model is used to evaluate the thermal hysteresis. More interestingly, it is found that the lowest thermal hysteresis is achieved at 10.4 at.% for Cu-doped NiTi; whereas the thermal hysteresis increases with increasing of Zr. The electronic structures of austenite phase are also discussed in detail.

## Introduction

Among the reported shape memory alloys (SMAs), NiTi alloys are the most attractive ones due to their unique shape memory effect and excellent superelasticity, resulting from the reversible thermoelastic martensite transformation (MT) between the high-temperature austenite phase and low-temperature martensite phases^1^. NiTi SMAs have been studied extensively and widely used for a variety of applications in a number of different fields, such as aeronautic, microelectronic and biomedical industries, since 1960s when first discovered by Buehler and Wiley^2^. However, some applications of the NiTi SMAs are greatly limited by its particular hysteresis (Δ*T*) and the martensite transformation temperature, *T*_*m*_, is usually lower than 100 °C^3^. The need of addressing these limitations has led to numerous experimental and theoretical researches^4–15^.

The addition of Fe and Co (substituted for Ni) or Al, V, Cr and Mn (substituted for Ti)^4^ depresses the *T*_*m*_ of NiTi alloys severely even at very small levels, but still results in a monoclinic martensite phase after MT. According to the recent reports^5,6^, there exists a strain glass (STG) transition in doped Ti_50_(Ni_50−*x*_D_*x*_) (D = Co, Cr, Mn) alloys. Wang *et al*.^7^ discovered the existence of strain glass in Ti_50_Ni_50−*x*_Fe_*x*_, beyond a critical Fe doping level *x* > *x*_c_ (5 < *x*_c_ < 6). Alloying NiTi with Cu (Cu substitutes for Ni) reduces the thermal hysteresis^8^ and composition sensitivity of the transformation temperatures^9^, meanwhile, changes the MT pathway^8,16,17^.

Transformation temperature increases linearly with the X content for alloys containing greater than approximately 10–15 at.% in ternary NiTi-X (X = Zr, Hf, Pd and Pt) SMAs^10^. These high-temperature shape memory alloys (HTSMAs) with the *T*_*m*_ higher than 100 °C have various potential applications. NiTiZr system has drawn much attention owing to the much lower cost compared with the noble metals (Pd, Pt, and Au). Experiments have indicated that, the *T*_*m*_ decreases with small amounts of Zr (X ≤ 3 at.%)^18^, and then sharply increases when Zr content exceeds 10 at.%, approaches as high as 170 °C for 20.2 at.% Zr^11–13^; martensite structure remains monoclinic (B19′)^11,12^; and its hysteresis is slightly wider than that of TiNi binary alloy^13^. Frenzel *et al*.^19^ found the strong dependence of *T*_*m*_ on alloy composition in binary NiTi, ternary Ni–Ti–X (X = Cr, Cu, Hf, Pd, V, Zr) and quaternary Ni–Ti–Cu–Y (Y = Co, Pd) SMAs.

From above experimental researches, it can be seen that the alloying elements play an important role in determining the phase transformation temperature, hysteresis as well as STG. Zarinejad and Liu^20^ conclude that the valence electron concentration is found to be an important parameter influencing the *T*_*m*_. There requires a better understanding of how they affect the phase structure and properties and the strong composition dependence of *T*_*m*_.

The first-principles has been used to study the microscopic origin of NiTi MT further. Recently, the site preference for ternary additions in NiTi has been systematic calculated^21^. The martensite crystal structures of Ti_50_Ni_50−*x*_Cu_*x*_ SMAs were investigated by using CASTEP code^22^. Wang’s ab-initio calculations^6^ show the origin of STG transition in Ti_50_(Ni_50−*x*_D_*x*_) (D = Cr, Mn, Fe and Co) alloys, and indicate that Cu and Pd cannot induce the STG transition. Hu *et al*.^14^ investigated the alloying effect of Zr on the elastic properties of Ni-rich Ni–Ti–Zr HTSMAs. Our previous work^15^ has investigated the effect of Pd on the martensitic transformation of Ni–Ti–Pd HTSMAs through DFT calculations. Attracted by the small hysteresis width^8^ and the low composition sensitivity of *T*_*m*_^9^ in Ni–Ti–Cu alloys, the relatively low materials cost and large change of *T*_*m*_ in Ni–Ti–Zr HTSMAs^10–13^. Consequently, we devote this paper to study the phase stability and transition behaviors of Ni–Ti–Cu and Ni–Ti–Zr alloys based on DFT, further rationalize the experimental findings about MT, and explain the strong content dependence of the phase stability of martensite crystal structure, transformation temperature and thermal hysteresis.

Generally, the DFT calculations are implemented at the temperature of 0 K^23^. As we are just concerned with the relative variations of the energy between austenite and martensite phases, the energy differences are almost unaffected by temperature. Transmission electron microscopy shows the presence of precipitates in Ni–Ti–Cu films after aging, precipitations vary with different annealings^24^. The effect of precipitation is beyond of the scope of the current work.

## Computational Models and Methods

**Figure 1:**
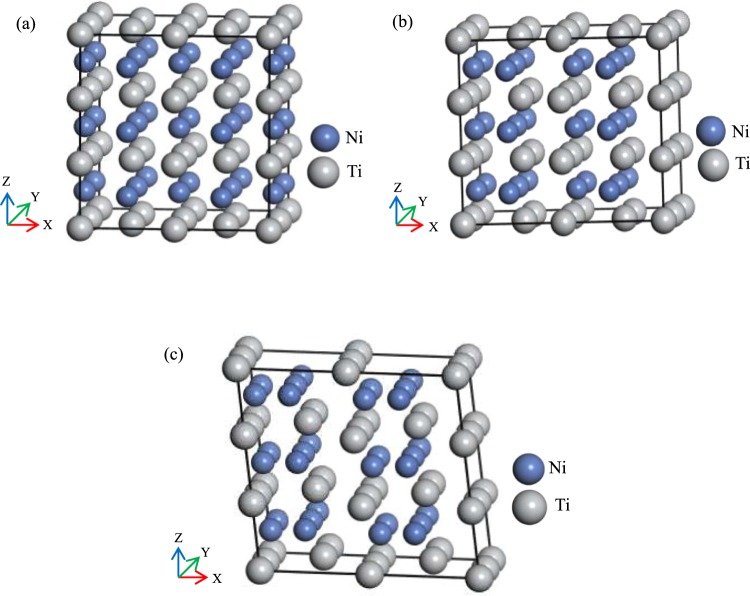
Schematic diagram of the 2 × 2 × 3 supercell model of austenite and martensite phase of NiTi alloy: (**a**) B2, (**b**) B19 and (**c**) B19′. Smaller blue spheres are Ni atoms; larger gray spheres are Ti atoms.

We focus only on the Ti-deficient Ni–Ti–Zr and Ni-deficient Ni–Ti–Cu alloys^4^, thus antisite defects were not discussed in the present work. In this paper, we adopt the Ti_50_(Ni_50−*x*_Cu_*x*_) and Ni_50_(Ti_50−*x*_Zr_*x*_) (*x* = 4.2, 6.3, 8.4, 10.4, 12.5, 18.8, 25) alloys as our modeling. Considering the symmetry of the cell, a 2 × 2 × 3 supercell containing 48 atoms of the austenite phase is chosen, and X, Y, Z directions are along [001]_*B*2_, ${[\bar{1}10]}_{B2}$ and [110]_*B*2_, respectively. The lattice parameters are ${\rm{a}}={a}_{0},\,{\rm{b}}={\rm{c}}=\sqrt{2}{a}_{0}$, here *a*_0_ is the lattice constants of cubic B2 phase. We focus on the orthorhombic B19 and monoclinic B19′ structures for the martensite phase observed experimentally in Ni–Ti–Cu and Ni–Ti–Zr shape memory alloys^11,12,16^. Figure 1 shows the supercell model of austenite and martensite phase of NiTi alloy. Geometry optimizations were implemented for all possible doping structures. More details of site preference for Ti_50_(Ni_50−*x*_Cu_*x*_) and Ni_50_(Ti_50−*x*_Zr_*x*_) alloys were shown in Supplementary Fig. S1, Supplementary Tables S1 and S2. The site occupation of B19 and B19′ phase corresponds to that of B2.

We calculate the most stable position of B2 phase with the principle of lowest energy first, and the site occupation of doped atoms of B19 and B19′ phase is set according to the B2. The energy difference between B2, B19 and B19′ phase is almost unaffected by the doping position. Moreover, the calculated deviations of energy of all possible doping position is about 0.2 eV.

The calculations are performed using DFT as implemented in the Vienna *Ab initio* Simulation Package^25–27^ (VASP) together with plane-wave projector augmented wave^28^ (PAW) pseudopotentials and the PW91^29^ generalized gradient approximation^30^ (GGA) for the exchange and correlation effects. The valence electron configurations for Ni, Ti, Cu and Zr are 3d^8^4s^2^, 3d^2^4s^2^, 3d^10^4s^1^ and 4d^2^5s^1^, respectively. We tested k-point sampling^31^ and an energy cutoff convergence for all supercells. As a result of the convergence tests, Brillouin zone sampling was performed using 4 × 4 × 4 special k-point mesh, the plane-wave cutoff energy was set to 500 eV. In the calculations, the total energies were converged up to 10^−4^ eV/atom, and the atomic positions in our models were fully relaxed until the force of every atom was less than 0.01 eV/Å. We relaxed the structure with ISIF = 7 option (not change cell shape, change cell volume and not relax ions) first, and then, with ISIF = 2 option (not change cell shape, not change cell volume and relax ions) in VASP.

Structural parameters adopted in the present work are summarized in Table 1. The calculated lattice parameters are in line with each other and with the experimental result.

**Table 1:** Lattice constants of B2, B19 and B19′ phases in NiTi (taken from our previous calculation ref^15^), compared with the experimental data and other ab initio calculations.

	B2				B19			B19′			
	Our calc.^15^	Exp.^42^	Other calc.^43,44^		Our calc.^15^	Other calc.^43,44^		Our calc.^15^	Exp.^45^	Other calc.^43,44^	
*a*(Å)	3.004	3.015	3.009	3.011	2.737	2.776	2.810	2.915	2.898	2.929	2.847
*b*(Å)	4.248	4.264	4.255	4.258	4.239	4.221	4.189	4.083	4.108	4.048	4.116
*c*(Å)	4.248	4.264	4.255	4.258	4.637	4.631	4.707	4.649	4.646	4.686	4.672
*β*(°)	90	90	90	90	90	90	90	98.26	98	97.8	97.78

## Results and Discussion

### Effects of additions (Cu or Zr) on phase stability of ternary NiTi-based alloys

**Figure 2:**
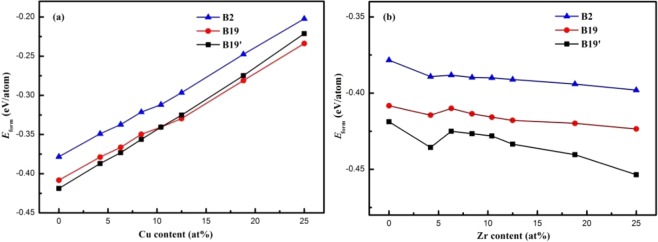
Formation energies, *E*_*form*_, as a function of the addition content in quasi-equiatomic ternary (**a**) Ti_50_(Ni_50−*x*_Cu_*x*_) and (**b**) Ni_50_(Ti_50−*x*_Zr_*x*_) alloys. The trendline is a fit through all data points.

In order to analyze the effect of Cu or Zr additions on phase stability, we evaluate the formation energies (*E*_*form*_) for B2, B19 and B19′ phases of Ti_50_(Ni_50−*x*_Cu_*x*_) and Ni_50_(Ti_50−*x*_Zr_*x*_) alloys, respectively. *E*_*form*_ is defined as the total energy of the alloy minus the concentration weighted average of the pure elements’ total energies at their equilibrium volumes^32–34^. As previously mentioned, the 2 × 2 × 3 supercell of B2, B19 and B19′ structures of Ti_50_(Ni_50−*x*_Cu_*x*_) and Ni_50_(Ti_50−*x*_Zr_*x*_) alloys that contains 48 atoms are constructed. It is calculated (per atom) as

1 $${E}_{form}^{1}=\frac{{E}_{tot}-[24E\,{{(}_{Ti}}^{hcp})+(24-n)\,E\,{{(}_{Ni}}^{fcc})+nE\,{{(}_{Cu}}^{fcc})]}{48}$$

2 $${E}_{form}^{2}=\frac{{E}_{tot}-[24E\,{{(}_{Ni}}^{fcc})+(24-n)E\,{{(}_{Ti}}^{hcp})+nE\,{{(}_{Zr}}^{hcp})]}{48}$$

where ${E}_{form}^{1}$ and ${E}_{form}^{2}$ are for Ti_50_Ni_50−*x*_Cu_*x*_ and Ni_50_Ti_50−*x*_Zr_*x*_ alloys respectively, *E*_tot_ is the total energy of Ti_50_Ni_50−*x*_Cu_*x*_ and Ni_50_Ti_50−*x*_Zr_*x*_ alloys, $E\,{{(}_{Ni}}^{fcc})$, $E\,{{(}_{Ti}}^{hcp})$, $E\,{{(}_{Cu}}^{fcc})$ and $E\,{{(}_{Zr}}^{hcp})$ denotes, respectively, the energy of one Ni, Ti, Cu and Zr atom in their bulk states, *n* is the number of doping atoms (Cu or Zr) in the supercell. The calculated results of *E*_*form*_ as a function of doping concentration are plotted in Fig. 2.

*E*_*form*_ of B2 phase is much higher than that of B19 and B19′ phases in NiTi alloy (*x* = 0), and, *E*_*fB*19′_ < *E*_*fB*19_ < *E*_*fB*2_ < 0, which reveals that the B19 and B19′ phases are more stable compared with the B2 phase, and, B19′ phase is the lowest temperature phase, which agrees with the experimental results^16^.

The *E*_*form*_ of each phase increases almost linearly with the increasing of Cu content which indicates that the phase stabilities of three structurers become worse (see Fig. 2(a)). This is in accordance with the experimental results^17^. There is a crossover at the formation energy curve for Cu = 10.4 at.% concentration. For Cu < 10.4 at.%, the B19′ phase is more stable than B19 phase; for Cu > 10.4 at.%, the B19 phase is more stable than B19′ phase, which indicates the phase transition path changed. For Cu < 10.4 at.%, phase transition takes place from B2 → B19′, while for Cu ≥ 10.4 at.%, the MT path thus becomes B2 → B19. From the calculation of formation energies, the most stable martensitic phase was changed from monoclinic to orthorhombic (see Fig. 2(a)), which is also in accordance with previous experimental works^8,16^.

For the Zr-doped, however, the formation energies of three phases decrease with the doping concentration, which means the stability for all phases increases with Zr doping. Moreover, the formation energy of B19′ is the lowest one. The B19′ phase is the most stable structure all the time with increasing *x*, which consistent with the previous results^11,12^, see Fig. 2(b).

**Figure 3:**
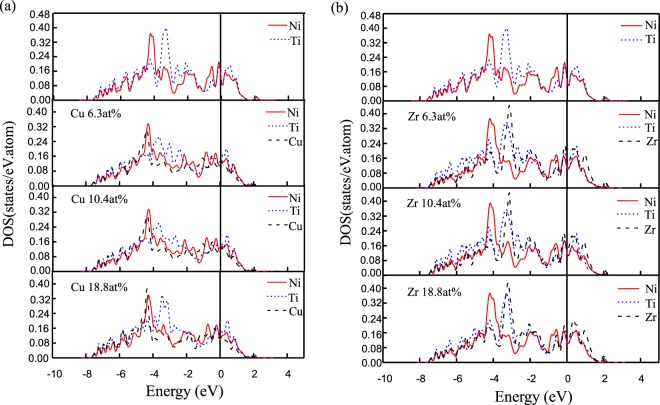
Partial density of states (PDOSs) of the doping atoms as well as their nearest Ti and Ni atoms in the austenitic phase of different doping concentration for (**a**) Ti_50_(Ni_50−*x*_Cu_*x*_) and (**b**) Ni_50_(Ti_50−*x*_Zr_*x*_) alloys. The vertical line denotes the Fermi level, which is located at 0 eV.

The partial density of states (PDOSs) of the austenitic phase with different doping concentration for Ti_50_(Ni_50−*x*_Cu_*x*_) and Ni_50_(Ti_50−*x*_Zr_*x*_) alloys have been calculated and plotted in Fig. 3. We can see that the PDOSs of Ni and Ti atoms have a sharp peak located at −4.4 and −3.5 eV, respectively, for pure NiTi alloy. The PDOSs of Cu atoms are similar to the Ni atoms, and, there is no obvious hybridization with the nearest neighbor Ti atoms. Cu doping weakens the overlap between Ti and Cu electronic states at −4 ~ −1.5 eV. With the increasing of the Cu concentration, the peak positions of Ti atoms and Ni-Cu resonated atoms shift to the higher energy slightly. This suggests that B2 phase become unstable with the increasing of Cu content, in accordance with the results of *E*_*form*_ (see Fig. 2(a)).

It can be seen from Fig. 3(b) that due to the Zr doping, the PDOSs of Zr atoms are similar to the Ti atoms, and there is obvious hybridization with the nearest neighbor Ti and Ni atoms. With the increase of Zr addition, there are the increasing resonant states between the Zr and its nearest neighbor Ti atoms at energy region −4 ~ −3 eV, which enhances the interaction between Ti and Zr atoms. The peak of Ni atoms and Ti-Zr resonated atoms shift to the lower energy slightly. This indicates that strong bonding states and thus increases the stability of the austenitic phase, which is in consistent with the formation energy result shown in Fig. 2(b).

### Composition dependence of transformation temperature in Ti_50_(Ni_50−*x*_Cu_*x*_) and Ni_50_(Ti_50−*x*_Zr_*x*_) alloys

The transformation behaviors can be described by the energy differences (Δ*E*) between the austenitic B2 structure and martensitic B19 and B19′ structure, Δ*E* = *E*_*B*2_ − *E*_*B*19/*B*19′_. Table 2 summarizes the energy differences for all three phases in NiTi. The calculated results indicate that B19 phase is lower in energy than B2 by 29.95 meV/atom, and, the monoclinic B19′ phase is the lowest in energy (Δ*E* = *E*_*B*2_ − *E*_*B*19′_ =  40.39 meV/atom) in NiTi alloys. The present calculations are in good accordance with other DFT studies.

**Table 2:** Computed energy differences Δ*E* = *E*_*B*2_ − *E*_*B*19/*B*19′_ (in meV/atom) in binary NiTi, compared with other first-principles studies.

Δ*E*	Present calc.	Other calc.^43^	Other calc.^44^
*E*_*B*2_ − *E*_*B*19_	29.95	30.07	28.57
*E*_*B*2_ − *E*_*B*19′_	40.39	41.91	39.46

**Figure 4:**
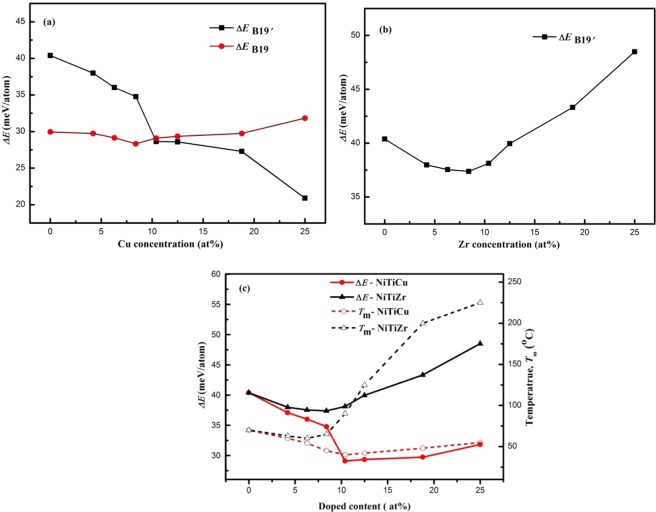
Dependency of the energy differences between austenite and martensite Δ*E* for (**a**) Ti_50_(Ni_50−*x*_Cu_*x*_) and (**b**) Ni_50_(Ti_50−*x*_Zr_*x*_) on doped content (0–25 at.%). (**c**) Composition dependence of Δ*E* and transformation temperature *T*_*m*_ of Ti_50_(Ni_50−*x*_Cu_*x*_) and Ni_50_(Ti_50−*x*_Zr_*x*_) alloys. The values of *T*_*m*_ refer to the right axis and those of Δ*E* to the left axis. *T*_*m*_ of Ni-Ti-Cu is cited from refs^16,35^, *T*_*m*_ of Ni-Ti-Zr is cited from refs^10,13^. Lines are guides for the eyes.

The previous observation^32^ shows that martensite transformation temperature is closely related to the value of Δ*E*, and, *T*_*m*_ usually increases with increasing Δ*E*. Figure 4 presents the concentration dependence of Δ*E* for the Ti_50_(Ni_50−*x*_Cu_*x*_) and Ni_50_(Ti_50−*x*_Zr_*x*_) alloys. For Ti_50_(Ni_50−*x*_Cu_*x*_) alloys with low doping concentrations (Cu < 10.4 at.%), as 0 < Δ*E*_*B*19_ < Δ*E*_*B*19′_(see Fig. 4(a)), the most stable phase is B19′ phase. The Δ*E*_*B*19′_ decreases slightly with increasing *x* (see Fig. 4(a)), thus the lower energy is needed for the martensitic transformation. The lower Δ*E* corresponds to a lower *T*_*m*_, therefore, the *T*_*m*_ decreases slightly. At high doping concentrations (Cu > 10.4 at.%), as 0 < Δ*E*_*B*19′_ < Δ*E*_*B*19_, the most stable phase is B19, and Δ*E*_*B*19_ increases slightly with increasing Cu content, thus a little higher *T*_*m*_ occurs.

It is clearly seen from Fig. 4(b) that, for Zr < 10.4 at.%, Δ*E*_*B*19′_ decreases slightly; with *x* further increasing, Δ*E*_*B*19′_ increases significantly. Lager Δ*E* indicates more energy is needed for the martensitic transformation thus corresponds to a higher *T*_*m*_.

In Fig. 4(c), we plot the total energy differences between B2 and martensite phases (Δ*E*) as well as *T*_*m*_ as a function of the doping concentration of Ti_50_(Ni_50−*x*_Cu_*x*_) and Ni_50_(Ti_50−*x*_Zr_*x*_) systems. *T*_*m*_ of Ni–Ti–Cu is cited from refs^16,35^, and *T*_*m*_ of Ni–Ti–Zr is cited from refs^10,13^. Addition of Cu decreases the *T*_*m*_, and then increases slightly with further increasing of Cu content. This makes the *T*_*m*_ less sensitive to composition changes comparing with the binary NiTi alloys, which is in accordance with previous experimental works^8,9,16,17,35^. Zr addition raises the *T*_*m*_ obviously, which is fully in line with the experimental observations that the Zr additions increase the *T*_*m*_ dramatically^10,13^.

### Influence of Cu or Zr on the hysteresis

Hysteresis (Δ*T*) in shape memory alloys (SMAs) is the macroscopic presentation of the energy dissipation of the martensitic phase transformation, which plays an important role in their thermomechanical behavior. The geometric non-linear theory^36^ of martensite specifies several conditions for SMAs with extremely low hysteresis. The first condition $det(U)={\lambda }_{1}\,{\lambda }_{2}\,{\lambda }_{3}=1$, represents no volume change during phase transformation, where *U* is the transformation stretch tensor that maps the martensite lattice to the austenite lattice, det*U* is its determinant and *λ*_1_ ≤ *λ*_2_ ≤ *λ*_3_ are the ordered eigenvalues of U. The second condition *λ*_2_ = 1 represents phase compatibility between austenite and martensite. The transformation stretch tensor between a bcc austenite and one particular lattice correspondence variant of an orthorhombic martensite is^37,38^

3 $$U=[\begin{array}{ccc}\beta  & 0 & 0\\ 0 & \frac{\alpha -\gamma }{2} & \frac{\alpha +\gamma }{2}\\ 0 & \frac{\alpha +\gamma }{2} & \frac{\alpha -\gamma }{2}\end{array}].$$

Their values are entirely determined by the lattice parameters of the austenite (*a*_0_) and the orthorhombic martensite phase (*a, b, c*): ${\rm{\beta }}={{\rm{\lambda }}}_{1}=\frac{a}{{a}_{0}}$, ${\rm{\alpha }}={{\rm{\lambda }}}_{2}=\frac{b}{\sqrt{2}{a}_{0}}$, ${\rm{\gamma }}={{\rm{\lambda }}}_{3}=\frac{c}{\sqrt{2}{a}_{0}}$.

According to the above theory, Cui *et al*.^38^ discovered that there is a strong relationship between hysteresis (Δ*T*) and *λ*_2_, but not with det(U), in Ni–Ti–Cu and Ni–Ti–Pd thin-films. Zhang *et al*.^39^ further demonstrated the same correlation for bulk alloys of Ni–Ti–Au, Ni–Ti–Cu, Ni–Ti–Pd, and Ni–Ti–Pt. Low hysteresis with phase compatibility between austenite and martensite was investigated in Ti_50_(Ni_50−*x*_Pd_*x*_) system^37^. Recently, the direct correlation between thermal hysteresis and functional stability was demonstrated in binary (Ni-Ti), ternary (Ni-Ti-Cu, Ni-Ti-Pd) and quaternary (Ni-Ti-Cu-Pd) SMAs^40,41^.

**Figure 5:**
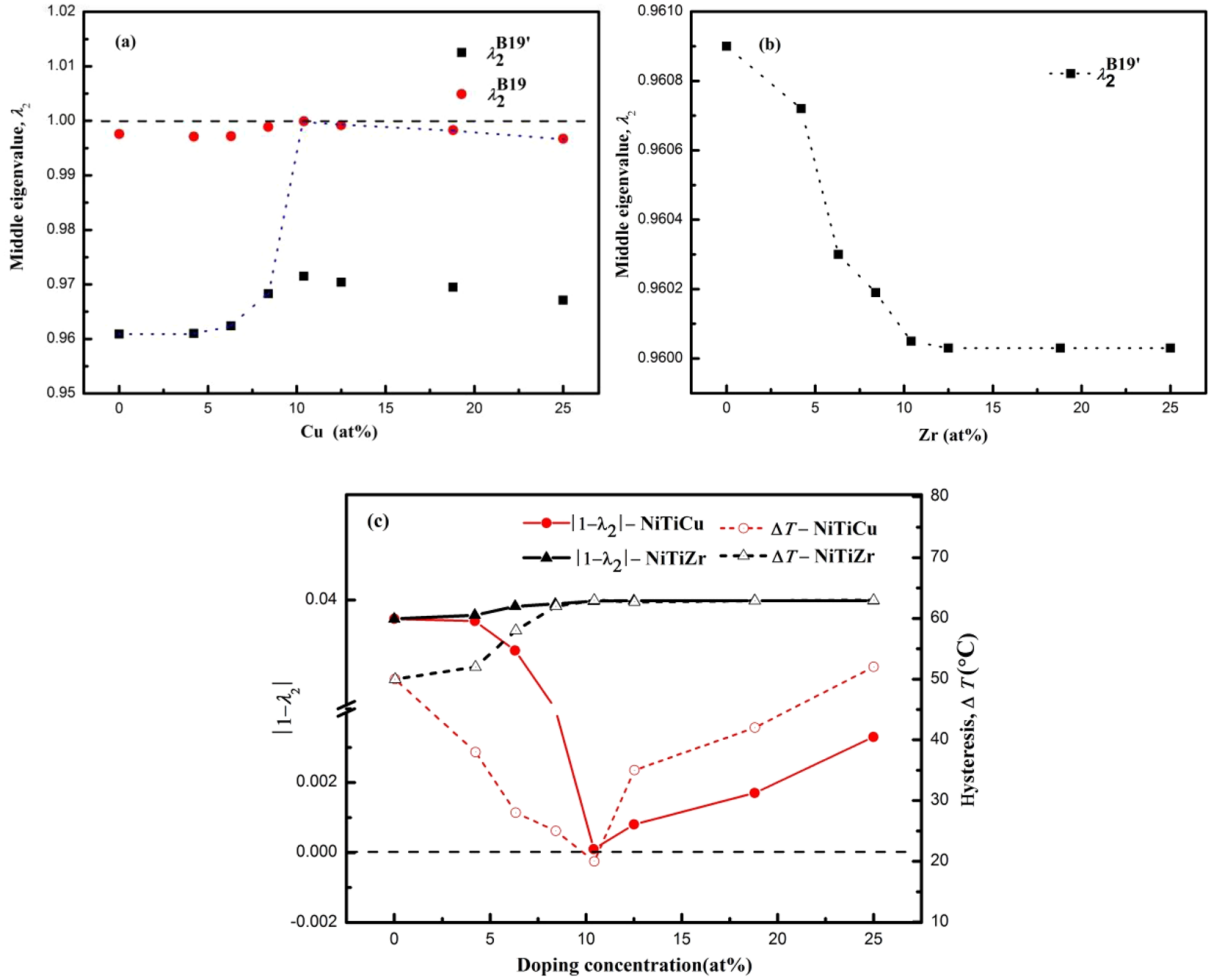
Middle eigenvalue *λ*_2_ vs. doped concentration of (**a**) Ti_50_(Ni_50−*x*_Cu_*x*_) and (**b**) Ni_50_(Ti_50−*x*_Zr_*x*_) alloys. (**c**) Effect of addition content on the |1 − *λ*_2_| and thermal hysteresis Δ*T* of Ti_50_(Ni_50−*x*_Cu_*x*_) and Ni_50_(Ti_50−*x*_Zr_*x*_) systems. The values of Δ*T* refer to the right axis and those of |1 − *λ*_2_| to the left axis. Δ*T* of Ni-Ti-Cu is cited from refs^10,13,19,35,38,39^. Δ*T* of Ni-Ti-Zr is cited from refs^10,13^.

To investigate the influence of alloy elements content on thermal hysteresis (Δ*T*) in the Ti_50_(Ni_50−*x*_Cu_*x*_) and Ni_50_(Ti_50−*x*_Zr_*x*_) systems, we calculated *λ*_2_ values by ${{\rm{\lambda }}}_{2}^{B19}={b}_{B19}/{b}_{B2}$ for B2 → B19 crystallographic transformation, *λ*_2_ shown as ${\lambda }_{2}^{B19^{\prime} }={b}_{B19^{\prime} }\,\sin \,{\rm{\beta }}/{b}_{B2}$ for B2 → B19′ phase transformation, where *b*_*B*2_, *b*_*B*19_ and *b*_*B*19′_ are the lattice parameters of the B2, B19 and B19′ structures in the [010] direction, respectively, and *β* is the monoclinic angle of the crystal structure of B19′ martensite. The calculated structural parameters of Ti_50_(Ni_50−*x*_Cu_*x*_) and Ni_50_(Ti_50−*x*_Zr_*x*_) for different doping concentration are listed in Supplementary Tables S3 and S4. The variations of *λ*_2_ vs. doping concentration for these alloys were shown in Fig. 5.

As indicated in Fig. 5(a), an increase in the percentage of Cu (substituted for Ni) increases the value of *λ*_2_, further approaching 1; for Cu content at 10.4 at.%, *λ*_2_ = 1; Cu > 10.4 at.%, *λ*_2_ is far from 1. In Fig. 5(c), |1 − *λ*_2_| was calculated in the present study while the corresponding Δ*T* values were taken from the literature^10,13,19,35,38,39^. One can see a remarkable collapse of the data onto two lines shaped like a V for Ti_50_(Ni_50−*x*_Cu_*x*_), and the widths of the thermal hysteresis have a minimum for |1 − *λ*_2_| approaches 0 (Cu = 10.4 at.%). *λ*_2_ quantifies the geometric compatibility of the martensite and the austenite, and the fit between the two phases increases as *λ*_2_ approaches 1^37–41^. The most stable martensite phase was changed from monoclinic to orthorhombic structure for Ti_50_(Ni_50−*x*_Cu_*x*_) alloys, which increases the phase compatibility, making *λ*_2_ approach 1. For other *λ*_2_ value which is lower or higher than 1, wider thermal hysteresis is observed. Adding Cu lower the width of the thermal hysteresis, Δ*T* appears to be minimized when Cu content at about 10.4 at.%at.%.

The value of *λ*_2_ decrease slightly for Zr < 10.4 at.%, and then nearly stable, keeping further from 1 (see Fig. 5(b)). This is because phase compatibility is reduced. With increasing of Zr content, *λ*_2_ deviates from 1 leading higher thermal hysteresis temperature for martensitic transformation. Therefore, replacement of Ti by Zr in the NiTi alloy raises the Δ*T* slightly in Ni_50_(Ti_50−*x*_Zr_*x*_) SMAs, which are in line with other literatures^10,13^.

## Conclusions

Our first-principles calculations on formation energies *E*_*form*_, energy differences between austenite and martensite Δ*E* and the middle eigenvalue of transformation stretch tensor *λ*_2_ for Ti_50_(Ni_50−*x*_Cu_*x*_) and Ni_50_(Ti_50−*x*_Zr_*x*_) systems allow us to draw the following conclusions. For Ti_50_(Ni_50−*x*_Cu_*x*_) alloys, when the doping Cu concentrations are less than 10.4 at.%, phase transformation takes place from B2 to monoclinic B19′ phase, while the doping concentration ≥10.4 at.%, phase transformation happens from B2 to orthorhombic B19 phase. According to the results of Δ*E* and *λ*_2_, for Cu < 10.4 at.%, adding Cu slightly decreases *T*_*m*_ and thermal hysteresis Δ*T*, moreover, gets a minimum of the widths of thermal hysteresis; with further doping, *T*_*m*_ and Δ*T* increase slightly. In short, the addition of Cu results in a decrease of *T*_*m*_ and Δ*T*. The present investigation exhibits that the replacement of Ti by Zr rises transformation temperature significantly, and increase the hysteresis slightly. Ni_50_(Ti_50−*x*_Zr_*x*_) alloy remains the B19′ martensitic phase, which leads the phase compatibility between austenite and martensite reduced and larger lattice deformation involved.

## Supplementary information

Supplementary materials

## Data Availability

The data that support the findings of this study are available from the corresponding authors upon reasonable request.
