# Supplementary material for: Martensitic transformation of Ti50(Ni50−xCux) and Ni50(Ti50−xZrx) shape-memory alloys
Source: Sci Rep. 2019 Mar 1;9:3221. doi: 10.1038/s41598-019-40100-z (PMC6397198; doi:10.1038/s41598-019-40100-z)
Supplement: Supplementary file 1 — Supplementary materials [file 41598_2019_40100_MOESM1_ESM.pdf]

Supplementary materials for

Martensitic transformation of $Ti_{50}(Ni_{50-x}Cu_x)$ and $Ni_{50}(Ti_{50-x}Zr_x)$ shape-memory alloys

Xiaolan Yang[1,2], Lei Ma[1] and Jiaxiang Shang[1,*]

[1]School of Materials Science and Engineering, Beihang University, Beijing 100191, China.

[2]School of Physics and Electronic Science, Zunyi Normal College, Zunyi 563002, China.

Corresponding Author

∗Email: shangjx@buaa.edu.cn (J. Shang)

Contents

Supplementary Figure S1...2

Supplementary Table S1 to S4...3

Supplementary Figure

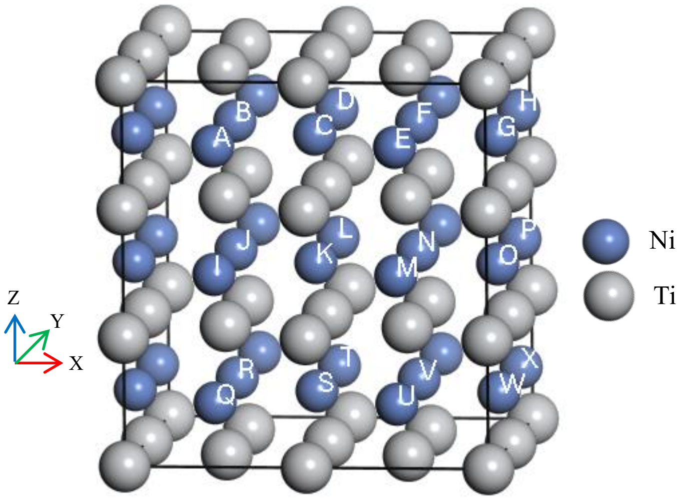

Supplementary Figure S1. Site occupation of Ni atoms in the 2 × 2 × 3 supercell model of austenite phase of NiTi alloy.

Supplementary Tables

Supplementary Table S1. The site occupation of Cu atoms in ternary $Ti_{50}(Ni_{50-x}Cu_x)$ shape memory alloys at different doping concentrations. n is the number of Cu in the supercell, accordingly, x can be acquired as $x = \frac{n}{48} \times 100$.

n	x	Cu
2	4.2	H, K
3	6.3	H, K, X
4	8.4	E, J, O, U
5	10.4	E, J, P, S, U
6	12.5	C, E, J, P, S, U
9	18.8	B, C, E, J, N, P, S, U, X
12	25	B, C, E, H, J, K, N, P, R, S, U, X

Supplementary Table S2. The site occupation of Zr atoms in ternary $Ni_{50}(Ti_{50-x}Zr_x)$ shape memory alloys at different doping concentrations. n is the number of Zr in the supercell, accordingly, x can be acquired as $x = \frac{n}{48} \times 100$.

n	x	Zr
2	4.2	H, K
3	6.3	E, J, U
4	8.4	E, J, O, U
5	10.4	E, J, O, U, S
6	12.5	F, H, I, K, V, X
9	18.8	B, C, E, J, M, P, S, U, X
12	25	B, C, E, H, J, K, M, P, R, S, U, X

Supplementary Table S3. Structural parameters of $Ti_{50}(Ni_{50-x}Cu_x)$ with different doping concentration. n is the number of Zr in the supercell, accordingly, x can be acquired as $x = \frac{n}{48} \times 100$.

n	x	B2				B19				B19'			
		a(Å)	b(Å)	c(Å)	V(Å^3)	a(Å)	b(Å)	c(Å)	V(Å^3)	a(Å)	b(Å)	c(Å)	V(Å^3)
0	0.0	3.004	4.248	4.248	54.19	2.737	4.239	4.637	53.79	2.915	4.083	4.649	55.35
2	4.2	3.010	4.256	4.256	54.53	2.739	4.244	4.643	53.99	2.920	4.090	4.658	55.61
3	6.3	3.013	4.261	4.261	54.70	2.743	4.249	4.648	54.17	2.922	4.101	4.661	55.83
4	8.4	3.016	4.265	4.265	54.86	2.751	4.260	4.661	54.61	2.925	4.129	4.666	56.31
5	10.4	3.020	4.271	4.271	55.09	2.758	4.270	4.667	54.96	2.926	4.149	4.667	56.63
6	12.5	3.021	4.277	4.277	55.24	2.766	4.274	4.671	55.22	2.929	4.150	4.672	56.77
9	18.8	3.029	4.283	4.283	55.55	2.770	4.275	4.681	55.43	2.934	4.152	4.695	56.99
12	25	3.041	4.299	4.299	56.20	2.774	4.285	4.691	55.76	2.949	4.158	4.703	57.63

Supplementary Table S4. Structural parameters of different doping concentration for $Ni_{50}(Ti_{50-x}Zr_x)$. n is the number of Zr in the supercell, accordingly, x can be acquired as $x = \frac{n}{48} \times 100$.

n	x	B2				B19'			
		a(Å)	b(Å)	c(Å)	V(Å^3)	a(Å)	b(Å)	c(Å)	V(Å^3)
0	0.0	3.004	4.248	4.248	54.19	2.915	4.083	4.649	55.35
2	4.2	3.021	4.272	4.272	55.15	2.932	4.105	4.676	56.24
3	6.3	3.034	4.291	4.291	55.87	2.942	4.120	4.692	56.84
4	8.4	3.043	4.303	4.303	56.33	2.952	4.131	4.708	57.39
5	10.4	3.049	4.312	4.312	56.87	2.964	4.140	4.727	57.99
6	12.5	3.061	4.329	4.329	57.36	2.973	4.156	4.742	58.56
9	18.8	3.086	4.363	4.363	58.74	2.994	4.189	4.775	59.87
12	25	3.109	4.397	4.397	60.11	3.024	4.221	4.876	62.22
